# Use of ringer's lactate solution does not eliminate the risk of strong ion difference related metabolic acidosis following on pump cardiac surgery

**DOI:** 10.1186/2197-425X-3-S1-A102

**Published:** 2015-10-01

**Authors:** I Norkiene, G Linkaite, J Guseinovaite, V Vicka, D Ringaitiene, T Jovaisa

**Affiliations:** Vilnius University, Clinic of Anaesthesiology, Vilnius, Lithuania; Vilnius University, Faculty of Medicine, Vilnius, Lithuania; Lithuanian University of Health Sciences, Clinic of Anaesthesiology, Kaunas, Lithuania

## Introduction

Perioperative use of Normal Saline is linked to hyperchloraemic or Strong Ion Difference (SID) related acidosis. It has been suggested, that this can be avoided by the use of balanced crystalloid solutions including Ringer's Lactate (RL). Significant changes in SID were noted previously in cardiac surgery, however recent publication disproved the link between SID and hydrogen ion concentration[1].

## Objectives

Our aim was to establish effects of routine use of RL on post-operative changes in SID and whether these changes result in metabolic acidosis. As pH is affected by both respiratory and metabolic components, we attempted to establish link between SID and base excess (BE), thus eliminating the potential compensatory effect of respiratory function.

## Methods

A retrospective analysis of 179 consecutive patients was conducted in tertiary referral university hospital. Approval of institutional ethics committee was obtained for this study. We analysed arterial blood gas samples at the point of patient arrival to ICU and 24 hours after surgery. Statistical analysis was performed using SPSS statistical package for Windows. Pearson correlation was used to establish the link between SID and BE. Paired samples T-test used to compare means between the two time-points.

## Results

Total volume of intravenous infusions in first 24 hours was 5865( ± 1073) ml, 95% of it was RL. 58 % of patients had metabolic acidosis with the BE of less than (-)2 mmol/L on arrival to ICU. There was significant correlation between SID and BE (p < 0.01). Significant improvement in metabolic acidosis was noted at 24 hours postoperatively, mean BE changed from (-)2.49 to 0.32 mmol/L (mean difference 2.8 mmol/L, p < 0.001). All of the improvement in BE is explained by change in SID from mean value of 31.0 to 34.2 mmol/L (mean difference 3.2 mmol/L, correlation significance of p < 0.001). Changes in SID were primarily driven by changes in serum sodium concentration that were threefold higher compared to those of chloride - 2.36 ( ± 2.6) mmol (p < 0.001) and 0.84 ( ± 3.2) mmol respectively (p = 0.01). There was no difference in pH between the two groups because of respiratory compensation at the first time-point resulting in significantly lower mean pCO2 of 41.3( ± 6.7) mmHg versus 43.6 ± 5.8 mmHg at 24 hours (p = 0.003).

## Conclusions

Our data confirms that there is a direct correlation between SID and BE following on pump cardiac surgery. Use of Ringer's Lactate prevented significant hyperchloraemia, but did not eliminate the risk of SID related metabolic acidosis. Change in SID was primarily linked to perioperative changes in serum sodium concentration.
